# Sex differences in caloric nystagmus intensity: Should reference values be updated?

**DOI:** 10.1111/nyas.15310

**Published:** 2025-03-06

**Authors:** Johannes Gerb, Sandra Becker‐Bense, Doreen Huppert, Konstanze Dunker, Valerie Schöb, Denis Grabova, Karoline Steinmetz, Ralf Strobl, Andreas Zwergal

**Affiliations:** ^1^ German Center for Vertigo and Balance Disorders (DSGZ) LMU University Hospital, LMU Munich Munich Germany; ^2^ Department of Neurology LMU University Hospital, LMU Munich Munich Germany; ^3^ Institute for Medical Information Processing Biometry and Epidemiology (IBE) LMU Munich Munich Germany

**Keywords:** gender medicine, neurotology, vertigo, vestibulo‐ocular reflex, vestibulopathy, water calorics

## Abstract

Bithermal caloric irrigation of the horizontal semicircular canals is a key method of neurotological diagnostics, allowing the detection of peripheral vestibular hypofunction in the low‐frequency range. Current diagnostic criteria for unilateral vestibulopathy (UVP), bilateral vestibulopathy (BVP), and presbyvestibulopathy (PVP) rely on gender‐neutral absolute or relative metrics. Here, we analyzed all bithermal water caloric examinations performed in the German Center for Vertigo and Balance Disorders (DSGZ) between 07/2018 and 01/2024 and calculated the total caloric reactivity (TR). Patient age and sex were collected as covariates. For UVP, BVP, and PVP diagnoses, international diagnostic criteria were applied. In total, 11,332 patients (6219 females, mean age 55.97±17.52 years) were included. Females displayed a higher TR (mean difference: 6.41°/s, *p*<0.001). The frequency of UVP, BVP, and PVP diagnoses based on *absolute* cut‐off values showed a significant male predominance (UVP: *n* = 1144, 548 females, odd ratio [OR] −0.32, *p*<0.001; BVP: *n* = 305, 138 females, OR −0.40, *p*<0.001; PVP: *n* = 813, 378 females, OR −0.37, *p*<0.001). However, the rate of UVP based on *relative* asymmetries showed no sex differences (*n* = 2971, 1595 females, OR −0.08, *p* = 0.06). Diagnostic criteria for UVP, BVP, or PVP, which utilize *absolute* caloric excitability cut‐offs, might need to be updated to address sex‐specific differences of caloric excitability.

## Graphical abstract

Bithermal caloric irrigation of the horizontal semicircular canals is a key method of neurotological diagnostics, allowing detection of peripheral vestibular hypofunction in the low‐frequency range. Current diagnostic criteria for unilateral vestibulopathy (UVP), bilateral vestibulopathy (BVP), and presbyvestibulopathy (PVP) rely on gender‐neutral absolute or relative metrics. Here, we analyzed all bithermal water caloric examinations performed in the German Center for Vertigo and Balance Disorders (DSGZ) between 06/2018 and 01/2024 and calculated the total caloric reactivity (TR). Patient age and sex were collected as covariates.

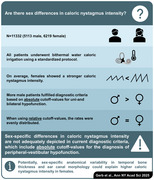

## INTRODUCTION

Since its first description by Robert Bárány in the early 20th century,^1^ bithermal caloric stimulation of the horizontal semicircular canals (i.e., consecutive flushing of the external ear canal with 30°C and 44°C water) and nowadays oculographic measurement of the maximum induced nystagmus (slow‐phase velocity, SPV in °/s) has represented the gold standard of neurotological laboratory testing for peripheral vestibular function in the low‐frequency range.^2^ Caloric nystagmus is assumed to be elicited through direct thermal stimulation of neuronal structures as well as thermally induced expansion of inner ear fluid compartments in the horizontal semicircular canal. This expansion is thought to induce ampullopetal movement of the endolymph with warm water irrigation, causing excitation and ipsilateral nystagmus through the deflection of sensory epithelia and subsequent activation of the vestibulo‐ocular reflex (VOR), and ampullofugal movement with cold water irrigation, causing inhibition and contralateral nystagmus. While direct thermal stimulation is independent of body position, the thermally induced endolymph convection is most pronounced in a supine position with a 30° head inclination, resulting in vertical alignment of the horizontal semicircular canals with the direction of gravity. By applying lower limits for the calorically induced nystagmus SPV, peripheral vestibular hypofunction in the sense of bilateral vestibulopathy (BVP^3^) or presbyvestibulopathy (PVP^4^) can be reliably diagnosed. Relative quantification metrics (typically Jongkees formula for side difference (SD): (((R30°+ R44°)—(L30°+ L44°)) / (R30°+ R44°+ L30°+ L44°)) × 100) are commonly used for diagnosis of unilateral vestibulopathy (UVP).^5^ Nowadays, bithermal caloric examinations depicting the low‐frequency range of the VOR are usually complemented with videooculographic head impulse testing (vHIT)^6^ in order to additionally capture its high‐frequency range^7^.

The current diagnostic criteria for BVP require bilateral caloric hypoexcitability, defined as the sum of warm and cold‐water stimuli not exceeding 6°/s maximum nystagmus SPV per side.^3^ For PVP in patients older than 60 years, a sum SPV between 6°/s and 25°/s SPV is required.^4^ These absolute SPV cut‐offs are the same for female and male patients and are based on older examinations not applying videooculography for the determination of nystagmus intensity.^8^ To the best of our knowledge, there are only two small‐sized (*n*<100) cohort studies that investigated caloric nystagmus intensity in healthy participants with respect to sex,^9^, ^10^ both observing no significant differences between male and female participants. However, an overall need for gender‐sensitive neurology^11^ and otology^12^ is now generally agreed upon. In fact, for certain aspects of vestibular physiology, sex differences have been reported (e.g., when testing the subjective visual vertical^13^, ^14^ or postural control^15^), and anatomical differences regarding vestibulocochlear nerve dimensions^16^ and myelination^17^ are evident (for an overview, see Smith et al.^18^).

Therefore, the aim of this study was to conduct a retrospective cohort analysis of all bithermal water caloric examinations performed in a large cohort of patients from a tertiary referral center, with the main focus on sex‐specific differences of caloric excitability, including side asymmetries. Additionally, we performed correlation analyses of mean caloric excitability with age in a subgroup with normal peripheral vestibular function according to diagnostic criteria.

## METHODS

### Patients

We collected all bithermal water caloric examinations conducted between 07/2018 and 01/2024 from the database of the German Center for Vertigo and Balance Disorders (DSGZ), Ludwig‐Maximilians‐Universität München, Germany. Age at the time of examination was calculated and patient biological sex was recorded for all patients.

### Diagnostics

All bithermal water caloric examinations were recorded with standardized hardware and software (EyeSeeCam, EyeSeeTech^19^) by experienced personnel. Before examination, contraindications for caloric irrigation, for example, perforations of the tympanic membrane, were ruled out by otoscopy. Water at 44°C was used for the warm water irrigations, while the cold‐water irrigations utilized a water temperature of 30°C. Caloric nystagmus was recorded using an infrared videooculography system. For comparison, in the BVP and PVP groups, additional data from video‐head impulse testing were used.

### Data processing

Only complete datasets, including warm (warm right, WR; warm left, WL) and cold (cold right, CR; cold left, CL) water irrigation on both ears, were included. For patients who underwent an additional 20°C water instillation on one or both ears, only the above‐mentioned standard temperature instillations were analyzed. Since the order of stimulation paradigms in previous studies had a variable impact on nystagmus intensity^20^, ^21^, all four measurements were analyzed individually by calculating the absolute values per stimulation paradigm (WR, WL, CR, CL). Additionally, we defined total reactivity (TR) by calculating the sum of absolute peak SPVs of all four paradigms. UVP, BVP, and PVP diagnoses were made according to the current diagnostic criteria of the International Classification of Vestibular Disorders by the Bárány Society^3^, ^4^, ^22^. For UVP, both relative unilateral deficits (i.e., side difference >30% in Jongkees formula with normal function on the contralateral side) as well as absolute unilateral deficits (i.e., unilateral absolute SPV<6°/s with normal function on the contralateral side) were analyzed. Additionally, a peripheral vestibular healthy controls cohort (pvHC) was defined by excluding all patients with manifest uni‐ or bilateral peripheral vestibular deficits. Note that this cohort still included PVP diagnoses (which per definition only occur in patients aged 60 and above), since an a priori exclusion of elderly patients with mild peripheral vestibular dysfunction would render any analysis of the age‐related decline of vestibular dysfunction impossible.

### Statistics

For further statistical analysis, JASP (jasp‐stats.org, version 0.18.3) and IBM SPSS (version 29.0.2.0) were used. To allow for unbiased analysis, in a first step, no a priori data filtering (e.g., BVP diagnoses) except for the removal of duplicates was performed. In a second analysis, BVP and UVP patients were excluded to calculate physiological values in subjects without lasting peripheral vestibular dysfunction (pvHC cohort). Occurrence rates were calculated using a Chi‐squared test.

Furthermore, in a data‐driven approach, sex‐dependent cut‐off values for BVP and PVP were defined based on the assumption of an equally balanced sex distribution of these disorders to achieve unbiased diagnosis rates without sex differences. To this end, the current cut‐off values (max. 6°/s per side for BVP, max. 25 °/s for PVP) were increased stepwise until a Chi‐squared test resulted in nonsignificant rate differences when applied to our cohort.

To determine age effects on TR, a correlation analysis (Spearman's rho) with the patient ages was performed, once with the full dataset and once with the pvHC cohort.

The study was approved by the data protection officer and the institutional review board of the Ludwig Maximilians‐Universität München (standardized database Dizziness Register, DIZZYReg no. 414–15), and all patients gave informed consent. The study was performed in accordance with the ethical standards laid down in the 1964 Declaration of Helsinki and its later amendments.

## RESULTS

### Sex differences

In total, caloric examinations from 11,332 patients (6219 females, 5113 males, mean age 55.97 ± 17.52 years) were included. Based on *absolute* caloric responses, 167 males (3.27% out of all male patients) and 138 females (2.22% out of all female patients) fulfilled the current diagnostic criteria for BVP, and 445 males (8.70% out of all male patients) and 388 females (6.24% out of all female patients) fulfilled the current criteria for PVP. Both BVP (♂: 54.72%; ♀:45.28%) and PVP (♂:53.42%; ♀:46.58) showed a male preponderance. Fisher's exact test to calculate the log odds ratio confirmed clear sex differences for the rate of BVP (*n* = 305, 138 females, OR −0.40, *p*<0.001) and PVP (*n* = 813, 378 females, OR −0.37, *p*<0.001). One thousand one hundred and forty‐four patients showed an *absolute* unilateral deficit (sum of warm‐ and cold‐water irrigation <6°/s on one side with normal function on the contralateral side; 596 males, 12.05% out of all male patients; 548 females, 9.01% out of all female patients). For the absolute values, Fisher's exact test showed a clear sex difference regarding the occurrence rate (*n* = 1144, 548 females, OR −0.32, *p* <0.001). Two thousand nine hundred and seventy‐one measurements showed a *relative* unilateral deficit (side difference >30% with normal function on the contralateral side in Jongkees formula; 1379 males, 27.89% out of all male patients; 1595 females, 26.18% out of all female patients). Fisher's exact test to calculate the log odds ratio (OR) of *relative* side asymmetry showed no significant sex difference (*n* = 2971, 1595 females, OR −0.08, *p* = 0.06).

In the complete cohort, females showed a stronger caloric response than males when we analyzed the absolute SPV values over all four caloric measurements together (TR) as well as separately (mean SPV TR ♀:♂ 67.12°/s ± 35.78°/s > 60.18°/s ± 33.20°/s; WL ♀:♂ 18.31°/s ± 13.37°/s > 16.72°/s ± 12.30°/s; WR ♀:♂16.47°/s ± 12.00°/s > 15.33°/s ± 10.97°/s; CL ♀:♂ 17.43°/s ± 11.04°/s > 15.06°/s ± 9.72°/s; CR ♀:♂14.92°/s ± 9.53°/s > 13.06°/s ± 8.51°/s). Statistical testing using the Mann−Whitney U test confirmed highly significant differences (TR: Wilcoxon‐test [W] 1.38×10^7^, *p*<0.001, HL −6.41°/s, *r* −0.11; WL: W 1.45×10^7^, *p*<0.001, Hodges‐Lehmann estimate [HL] −1.21°/s, rank biserial correlation [*r*] −0.06; WR: W 1.48×10^7^, *p*<0.001, HL −0.78°/s, *r* −0.05; CL: W 1.37×10^7^, *p*<0.001, HL −2.03°/s, *r* −0.12; CR: W 1.38×10^7^, *p*<0.001, HL −1.61°/s, *r* −0.11). The Mann−Whitney U test was chosen since the data was not normally distributed (test of normality and detrended Q‐Q‐plots in Supplementary Materials #1 and #2).

Even after post‐hoc removal of the UVP cases, specific differences between sexes were confirmed (TR: W 7.56×10^6^, *p*<0.001, HL −5.35°/s, *r* −0.09; WL: W 7.94×10^6^, *p*<0.001, HL −0.86°/s, *r* −0.05; WR: W 8.10×10^6^, *p* = 0.04, HL −0.45°/s, *r* −0.03; CL: W 7.38×10^6^, *p*<0.001, HL −1.79°/s, *r* −0.11; CR: W 7.29×10^6^, *p*<0.001, HL −1.70°/s, *r* −0.12). In a final analysis, only the pvHC cohort (e.g., the healthy subgroup) was analyzed: sex‐specific differences prevailed in all instillation paradigms except for WR (TR: W 7.20×10^6^, *p*<0.001, HL −4.83°/s, *r* −0.09; WL: W 7.58×10^6^, *p* = 2.41×10^−3^, HL −0.70°/s, *r* −0.04; WR: W 7.75×10^6^, *p* = 0.17, HL −0.29°/s, *r* −0.02; CL: W 7.03×10^6^, *p*<0.001, HL −1.66°/s, *r* −0.11; CR: W 6.94×10^6^, *p*<0.001, HL −1.59°/s, *r* −0.12; Figure 2).

The hypothetical application of data‐driven sex‐adapted absolute cut‐off values (Table 1) for UVP, BVP, and PVP resulted in a more equally distributed percentage of UVP, BVP, and PVP (UVP: 596 [12.05%] males, 731 [12.02%] females; BVP: 167 [3.31%] males, 181 [2.94%] females; PVP: 435 [8.63%] males, 513 [8.34%] females) without significant difference in Fisher's exact test (UVP: *n* = 1327, 731 females, OR −0.003, *p* = 0.98, BVP: *n* = 348, 181 females, OR −0.12, *p* = 0.27; PVP: *n* = 948, 513 females, OR −0.04, *p* = 0.61).

**TABLE 1:** Sex‐adapted new cut‐off values for bithermal water calorics suggested by a data‐driven approach based on the assumption of a balanced male/female ratio in BVP, PVP, and UVP.

		Male patients	Female patients
UVP	Sum of absolute SPV from warm and cold water caloric testing on one side	< 6°/s	< 8°/s
BVP	Sum of absolute SPV from warm and cold water caloric testing on both sides	< 6°/s	< 8°/s
PVP	Aged 60 years and above; sum of absolute SPV from warm and cold water caloric testing, per side	between 6°/s and 25°/s	between 8°/s and 30°/s

### Age effects

Patient age had no significant effects on overall TR (Spearman's rho −0.01, *p* = 0.18). When analyzing individual caloric paradigms, statistical significance was reached; however, the effect size was negligible (WL: Spearman's rho 0.04, WR: 0.07, CL: −0.05, CR: −0.12, all *p*<0.001). Note that these primary analyses were performed over all datasets without any a priori filtering or exclusion of, for example, BVP or PVP to depict a real‐world heterogeneous dataset. However, after the exclusion of BVP and UVP patients, statistical significance (albeit with a negligible effect size) was reached for TR as well as the individual caloric stimulations, showing an age‐related increase of TR and the warm water irrigation paradigms, and an age‐related decrease in the cold water paradigms (*n* = 8048; Spearman's rho TR: 0.04, *p* = 0.002, WL: 0.09, *p*<0.001; WR: 0.14, *p*<0.001; CL: −0.02, *p* = 0.05; CR: −0.11 *p* = 0.02). Based on this cohort, mean normative SPV values per age group were created (Figure 2 and Supplementary Material #3).

### Distribution of unilateral peripheral vestibular dysfunction

Analysis of *relative* unilateral deficits (*n* = 2971) showed a slight predominance of right‐sided deficits for both sexes, demonstrated by the relative proportion diverging significantly from 0.5 in a binomial test (with a value around 0.5 being the expected equal distribution; Figure 3). This effect was observable for males (*n* = 1379, right‐sided deficits: *n* = 796, proportion: 0.58, *p*<0.001) as well as females (*n* = 1592, right‐sided deficits: *n* = 960, proportion: 0.60, *p*<0.001). There was no side preference for *absolute* unilateral deficits (*n* = 1144) in males (*n* = 596, right‐sided deficits: *n* = 288, proportion: 0.48, *p* = 0.44), and a slightly right‐sided predominance in females (*n* = 548, right‐sided deficits: *n* = 298, proportion: 0.54, *p* = 0.04).

### Comparison with vHIT findings

Based on vHIT gain at 60 ms, 47.1% of BVP patients (i.e., values <0.6) were female, and 54.6% of PVP patients (i.e., values between 0.6 and 0.8) were female. No statistically significant sex effects were observable in binomial testing, demonstrated by the relative proportion of male and female patients not diverging significantly from 0.5. This could be shown in both BVP (proportion of male patients: 0.53, *p* = 0.22) and PVP (proportion of male patients: 0.45, *p* = 0.45).

## DISCUSSION

**FIGURE 1:**
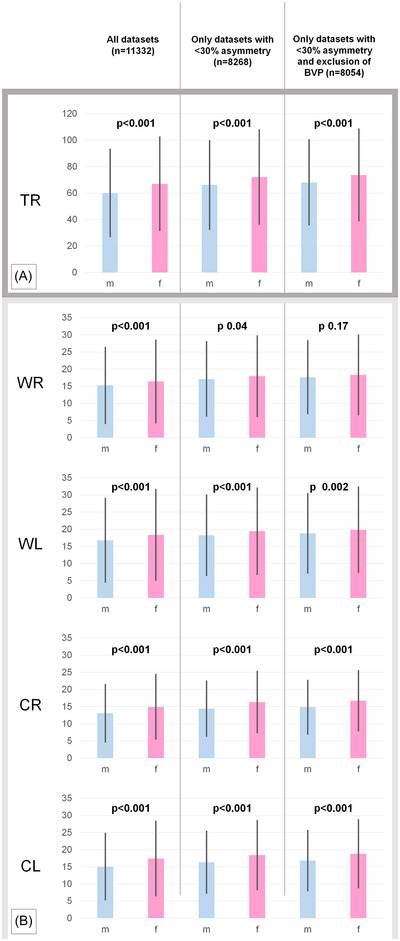
Absolute SPV [°/s] of caloric nystagmus (mean ± SD) from (1) all 11,332 datasets (left column), all datasets without UVP (middle column, *n* = 8268), and all datasets without UVP and without BVP (right column, *n* = 8054), divided by patient sex (*p*‐values from Mann−Whitney U test, error bars 95% CI). In all group analyses, a slightly stronger absolute caloric response in females was evident in the TR (calculated as the sum of all four irrigation paradigms (A), and in the individual irrigation paradigms (B)). Abbreviations: CL, cold caloric irrigation on the left side; CR, cold caloric irrigation on the right side; TR, total reactivity; WL, warm caloric irrigation on the left side; WR, warm caloric irrigation on the right side.

**FIGURE 2:**
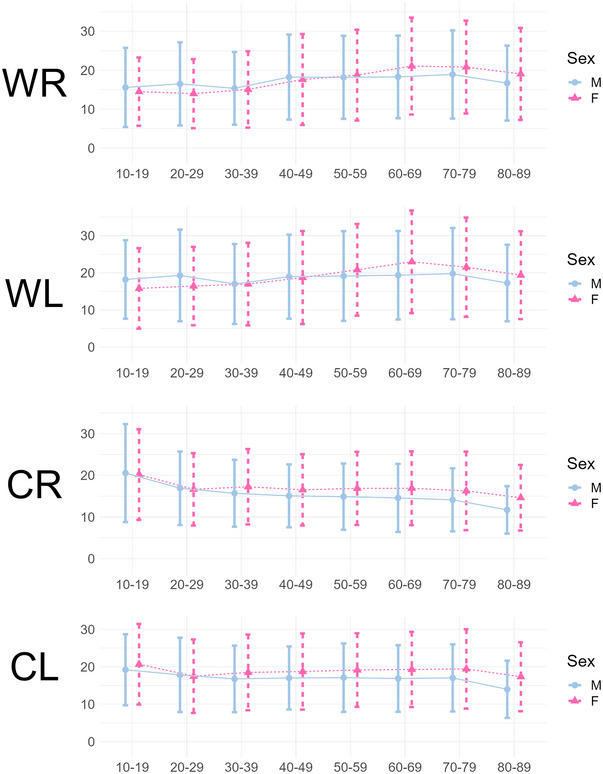
Mean SPV [°/s] ±SD per instillation paradigm in the pvHC cohort depicted by different ages between 10 and 90 years (*n* = 8016, age groups 0–9 and 90–99 not shown due to insufficient data), showing small age effects: a minor increase of caloric nystagmus in the warm water conditions and a small decrease in the cold‐water paradigms. However, overall, the caloric excitability remains remarkably stable until high age. Abbreviations: CL, cold caloric irrigation on the left side; CR, cold caloric irrigation on the right side; WL, warm caloric irrigation on the left side; WR, warm caloric irrigation on the right side.

In this large‐scale analysis of relative and absolute mean caloric nystagmus intensities, a significant sex difference was detected with females typically exhibiting slightly but significant stronger responses (Figure 1). This was also true in the pvHC, that is, the healthy subgroup after the removal of all patients with manifest uni‐ or bilateral peripheral deficits. Mean caloric nystagmus intensity showed no clinically relevant age dependency (Figure 2).

These general male/female differences could potentially result from sex‐dependent intrinsic characteristics of water stimulation of the external ear: first, female skulls on average exhibit less petrosal bone thickness compared to male skulls^23^, second, male external auditory canals are typically wider than in females^24^. While the exact mechanism of caloric nystagmus is still partially debated, for example, regarding the effect of gravity^25^, the role of thermal convection has been unequivocally clear. Theoretically, thinner petrosal bone would result in faster thermal convection in females compared to men, who typically exhibit thicker bones. Similarly, wider external auditory canals could reduce the amount of heat exchange, for example, if the instillation device is angled during the water instillation, or if the wider auditory canal allows for more water to flow outward before reaching the tympanic membrane. All of these mechanisms could result in an overall larger caloric effect on the labyrinth and stronger nystagmus intensity in women, but might only be found in larger cohorts, while individual differences might nullify this effect in smaller cohorts.

For the diagnoses of BVP and PVP based on the *absolute* SPV values, in fact, we found a male preponderance (BVP: 54.75% males; PVP: 53.51% males). One can speculate, whether these different rates are due to a factual male preponderance in BVP and PVP, as reported earlier^3^, or if they instead constitute a sign of underrepresentation of females in the current diagnostic criteria based on sex differences in caloric irrigation. For BVP, currently, a de facto sex bias with a higher prevalence in men is assumed. For example, Iwasaki et al. reported a slightly higher rate of male BVP patients (56.4%) in a Japan‐wide study^26^; similarly, Zingler et al.^27^ reported a male preponderance (62%) in Germany. A more recent study by Moyaert et al. using not only bithermal water caloric testings but also rotatory chair and/or vHIT testing, reported that 56% of BVP patients were male^28^. Mancino‐Moreira et al. reported a similar distribution (56.8% male) when using vHIT only^29^. All of these rates align with the findings in our cohort, in which 54.75% of BVP patients (using caloric criteria of the current diagnostic guidelines) were male. However, for BVP and PVP, the sex ratio was not significantly biased towards men when vHIT gain cut‐off criteria were used. Taking this aspect into account, at least some of the male preponderance for BVP and PVP may be assigned to a priori sex differences in caloric nystagmus intensity. In line with these considerations, in our study, UVP male preponderance was only seen with absolute but not relative readouts of caloric irrigation. Based on our findings, we suggest sex‐adapted absolute cut‐off values (Table 1) for UVP, BVP, and PVP, which reduce this male preponderance. Note that for the clinical diagnosis of peripheral vestibular deficits, other criteria still need to be fulfilled, for example, postural imbalance and oscillopsia for BVP^3^, or gait disturbance and chronic dizziness for PVP^4^. Here, further research is required to investigate if the clinical diagnosis rates of BVP and PVP with these sex‐adapted values still show effects of patient sex, such as a male preponderance in BVP.

No systematic correlation between patient age and total caloric reactivity was found in the overall cohort. However, for the individual paradigms, a small statistical significance was reached: both warm‐water instillations showed a slight increase, and the cold‐water instillations a slight decrease with higher age. This pattern prevailed when excluding UVP and BVP from the patient cohort and analyzing only healthy subjects in the pvHC cohort. Remarkably, the average caloric excitability remained relatively stable even with higher age; this contrasts with other human senses such as vision or hearing (e.g., presbyopy or presbyacusis), which show relevant systematic deterioration with aging.^30^, ^31^ Accordingly, previous research showed that isolated PVP is a very rare clinical entity, which usually needs to be accompanied by other multisensory dysfunctions to reach clinical relevance.^32^ At the current time, it is unclear why the caloric excitability to warm‐water instillations seems to increase with age, while the caloric excitability to cold‐water instillations seems to decrease. Potentially, aging effects on body temperature (with a slight decrease of average body temperature over the lifespan^33^) could result in a slightly higher temperature gradient in elderly patients during the warm‐water instillations, and a smaller temperature gradient during the cold‐water instillations. However, further research is necessary to deduce a clear relationship between patient body temperature and caloric excitability.

**FIGURE 3:**
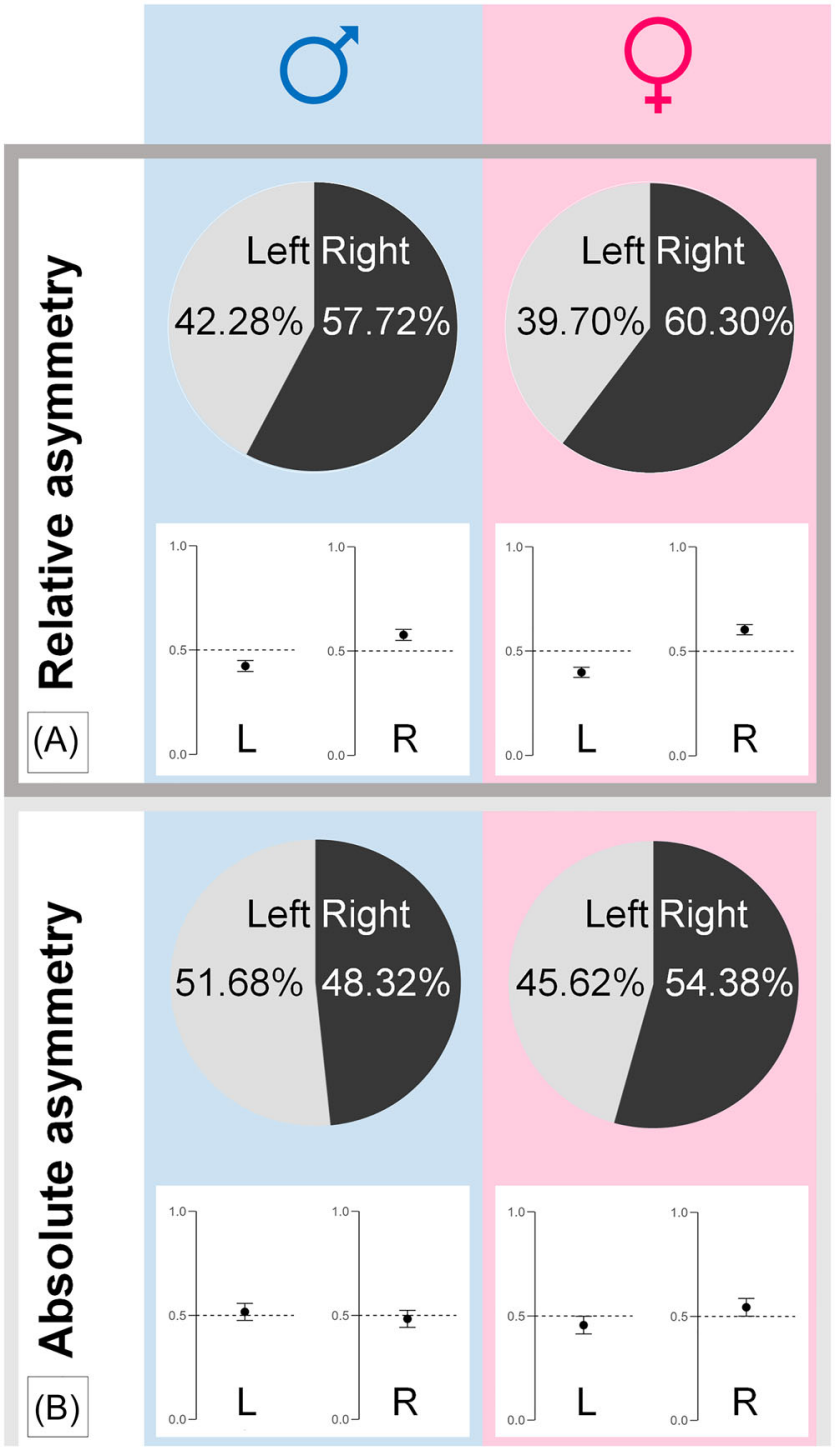
Pie charts and bar plots of side distribution of unilateral peripheral vestibular dysfunction, divided by sex (blue male, pink female). (A) Relative asymmetries (i.e., asymmetry >30% in the Jongkees formula, *n* = 2971) were more common on the right side independent of patient sex (binomial *t*‐test differing significantly from the expected symmetrical rate of 0.5). (B) For absolute unilateral caloric deficits (i.e., unilateral absolute SPV<6°/s with normal function on the contralateral side, *n* = 1144), a slight right‐sided predominance was found, but only in female patients.

As an additional finding, an overall right‐sided predominance of relative unilateral vestibular hypofunction was observed (Figure 3). For absolute values, this right‐sided predominance was only observed in female patients. Interestingly, a slight right‐sided predominance has been reported previously in small‐scale studies on patients with acute unilateral vestibulopathies/vestibular neuritis.^34^ Notably, in our cohort, further etiologies of unilateral vestibular hypofunction were included, for example, Menière's disease, vestibular schwannoma, or other peripheral vestibular disorders.

As a limitation, it should be noted that this study was conducted in patients who presented in our tertiary center because of vertigo and balance problems, and not in totally healthy subjects. Thus, the results might not be transferable to the general population. However, patients were carefully investigated and the subgroup analyses in the pvHC cohort, that is, patients without peripheral dysfunction such as uni‐ or bilateral high‐ and/or low‐frequency deficits, confirmed the results of the total cohort.

Despite comparable demographics regarding mean age or rate of caloric asymmetries, some vestibular disorders do show sex‐specific prevalence. For example, vestibular migraine is known to be more common in females by a factor of 2:1^35^ and can lead to increased caloric excitability^36^ as well as higher rates of subjective nausea and vertigo.^37^ However, this circumstance can only be a relevant confounder for the TR analysis (cf. Figure 1) but keeps the absolute rates of BVPs and PVPs unaffected.

Ultimately, it is crucial to avoid circular reasoning when discussing the question of sex differences in bithermal water caloric testing. Both a higher rate of acquired bilateral vestibular hypofunction in males as well as a higher average caloric excitability in females would result in the same clinical findings on a group level, namely, a slightly higher mean SPV in females as it is observed in this study. Only large enough healthy cohorts or population‐based averages might be able to finally provide the true reason for this observation. However, earlier cohorts^9^, ^10^ were drastically underpowered for disclosing sex differences compared to our study.

## CONCLUSION

In this large single‐center clinical cohort, sex differences on the basis of absolute caloric SPV intensity (with females exhibiting slightly stronger responses) were observed. This effect persisted even after the exclusion of BVP and UVP cases, that is, representing a subcohort with normal peripheral vestibular function. If this finding can be confirmed in sufficiently powered population‐based healthy subject datasets, diagnostic criteria for peripheral vestibular hypofunction based on absolute values (currently the case for UVP, BVP, and PVP) might need to be updated to better accommodate male and female patients. Total caloric excitability does not generally decrease with higher age.

## AUTHOR CONTRIBUTIONS

J.G.: Study conceptualization, study design, data acquisition, data analysis, statistical analysis, data visualization, drafting the manuscript, and revising the manuscript. S.B.‐B., D.H., and A.Z.: Study conceptualization, study design, study supervision, data analysis, data visualization, drafting the manuscript, revising the manuscript, and providing funding. K.D., V.S., D.G., and K.S.: Data acquisition, drafting the manuscript, and revising the manuscript. R.S.: Statistical analysis, drafting the manuscript, and revising the manuscript.

## COMPETING INTERESTS

The authors declare no competing interests.

## Supporting information



Supporting Information

## Data Availability

The data that support the findings of this study are available on request from the corresponding author. The data are not publicly available due to privacy or ethical restrictions.
