# Supplementary material for: Sex differences in caloric nystagmus intensity: Should reference values be updated?
Source: Ann N Y Acad Sci. 2025 Mar 6;1546(1):136–43. doi: 10.1111/nyas.15310 (PMC11998476; doi:10.1111/nyas.15310)
Supplement: Supplementary file 1 — Supporting Information [file NYAS-1546-136-s001.docx]

**Supplementary Material**

**#1: Normality testing from the distribution of absolute values for warm water caloric irrigation of the left ear (Wl); absolute values for warm water caloric irrigation of the right ear (Wr); absolute values for cold water caloric irrigation of the left ear (Cl); absolute values for cold water caloric irrigation of the right ear (Cr); the sum of all four paradigms (total reactivity, TR)**


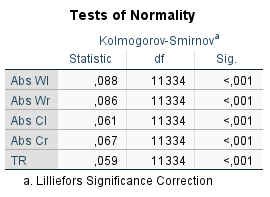


**#2: Histogram and detrended normal Q-Q-Plots per paradigm**

- 1. **Wl**


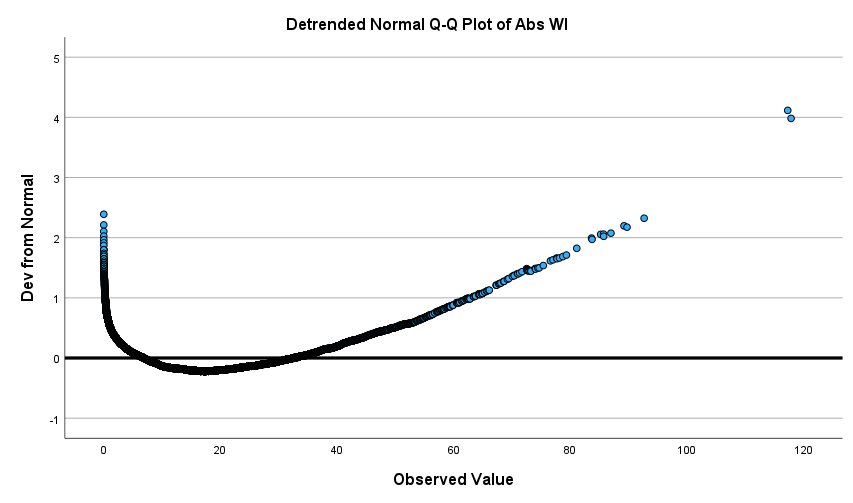

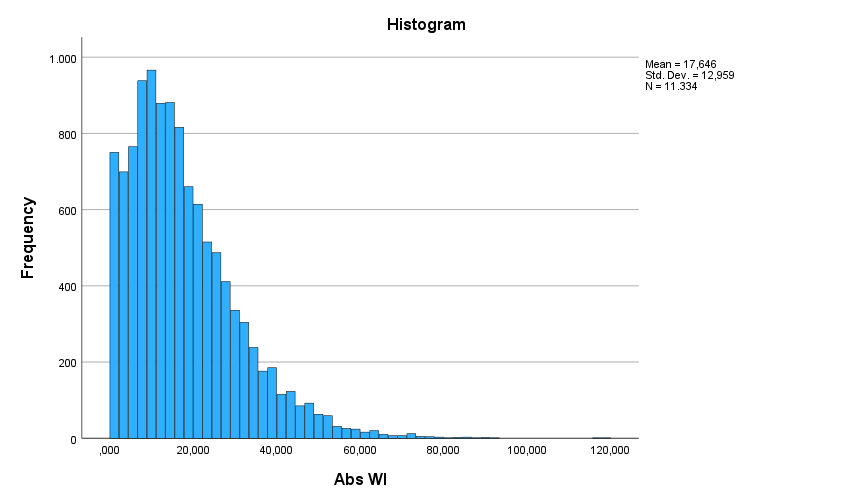


- 1. **Wr**


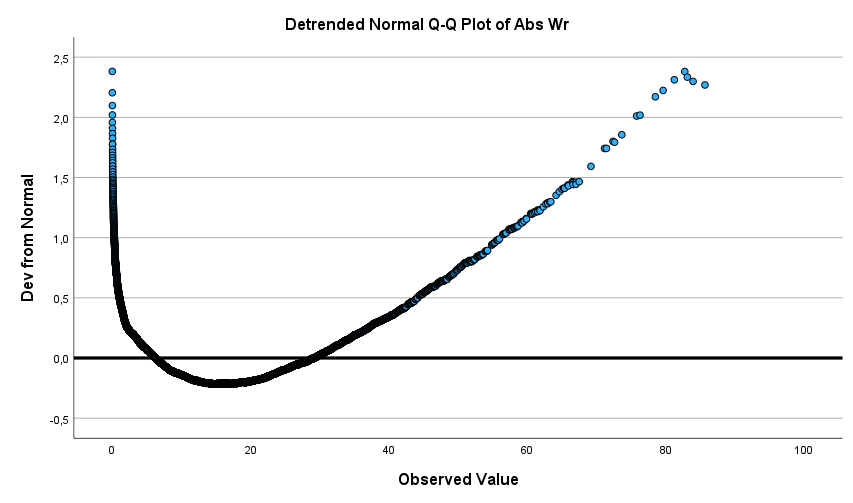

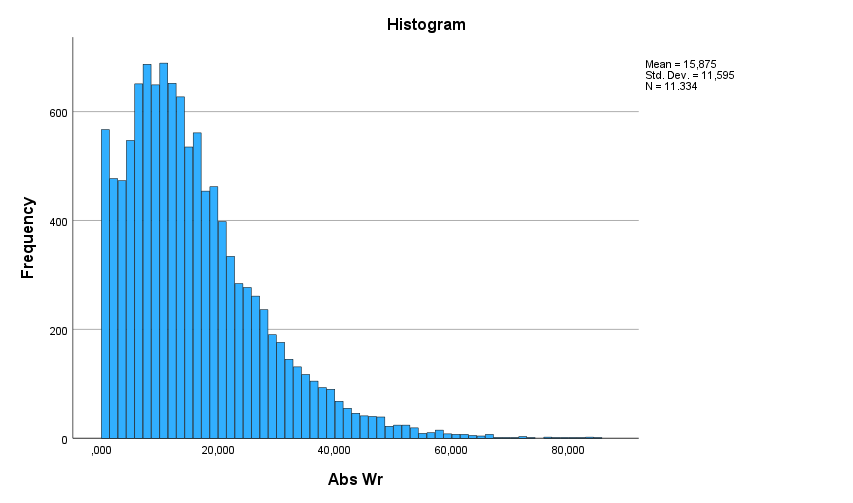


- 1.
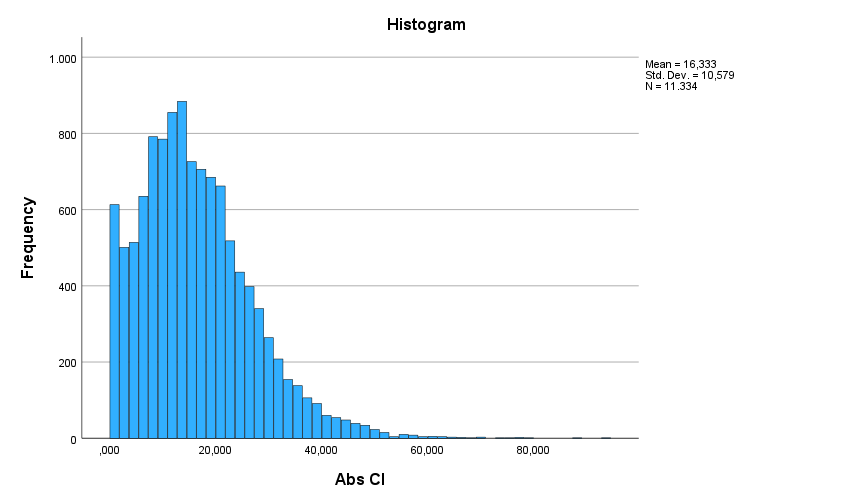
**Cl**


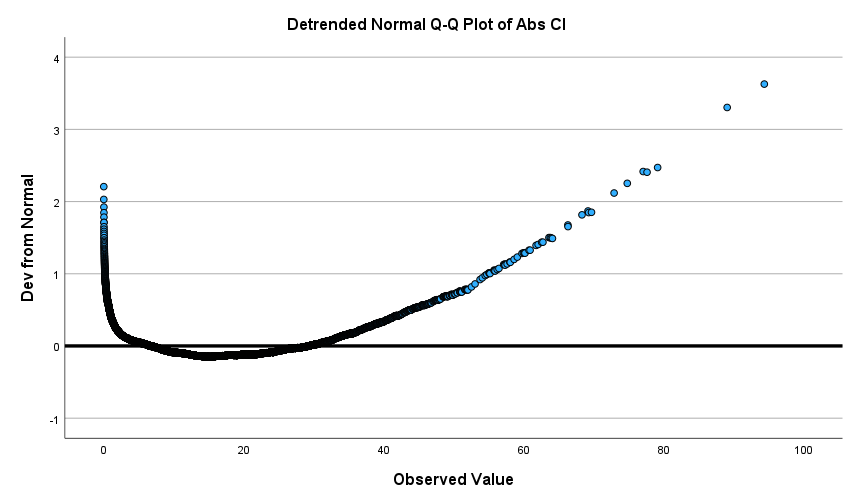


- 1.
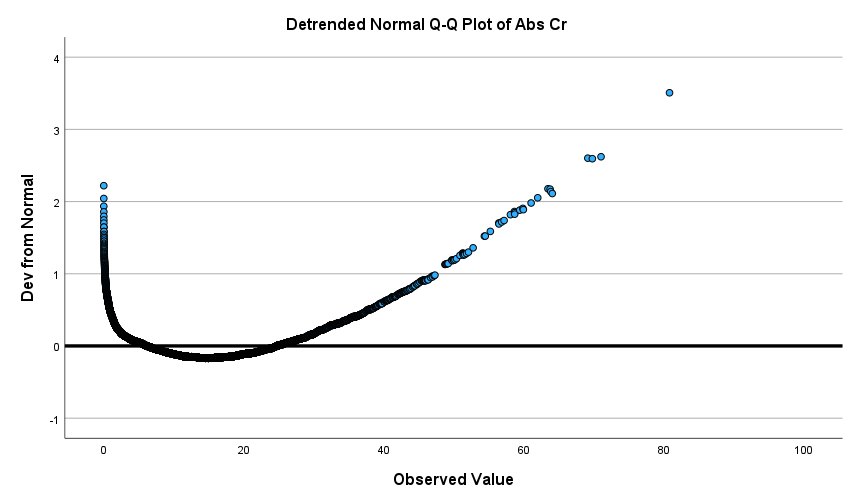

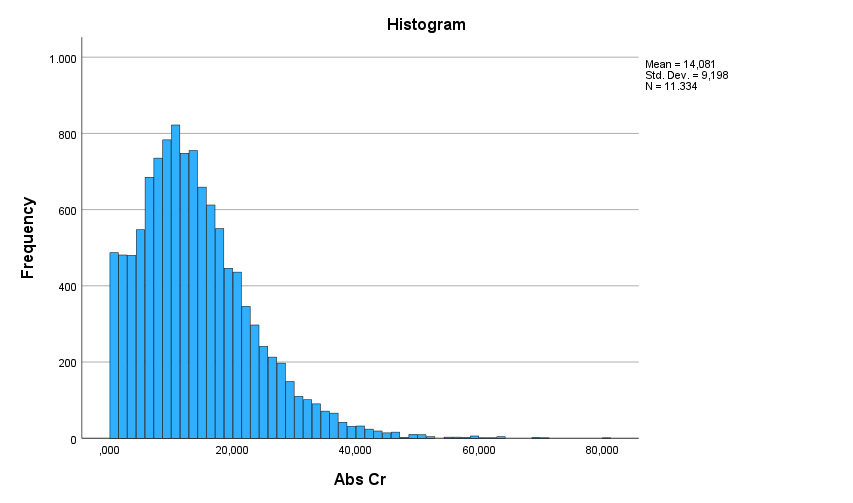
**Cr**
  2. **TR**


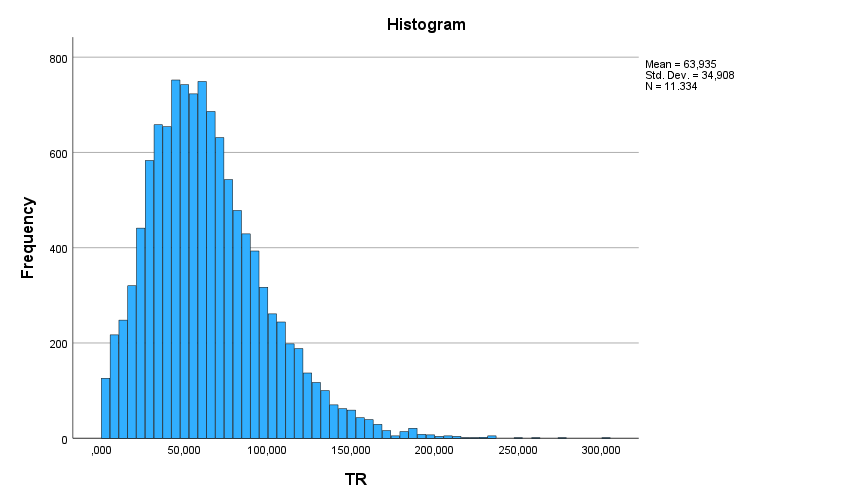

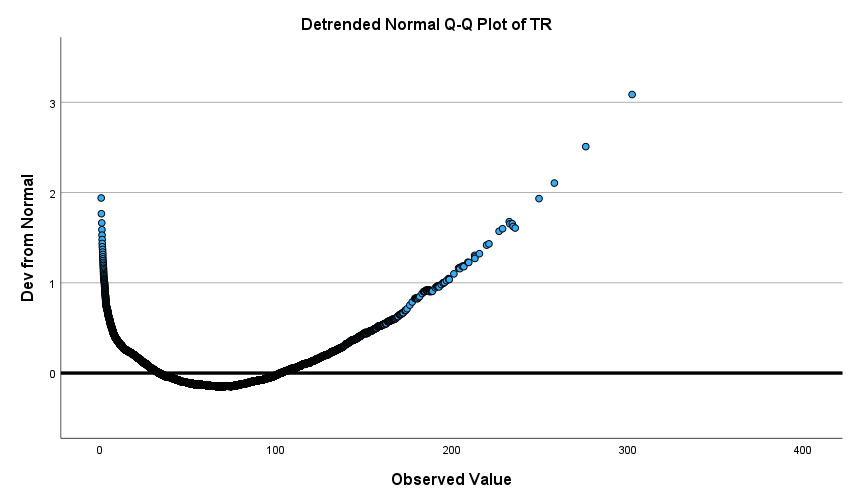


**#3: Normative data from caloric testing: mean absolute SPV values in °/s per patient age, grouped by decade. Patients aged below 10 and above 90 were excluded, since not enough datasets were available.**

| Age group |  | n | Warm water, left ear | Warm water, right ear | Cold water, left ear | Cold water, right ear |
| --- | --- | --- | --- | --- | --- | --- |
| 10-19 | f | 136 | 15.81 ± 10.84 | 14.51 ±8.77 | 20.66 ± 10.77 | 20.20 ± 10.87 |
|  | m | 70 | 18.20 ± 10.57 | 15.57 ± 10.17 | 19.19 ± 9.48 | 20.55 ± 11.75 |
| 20-29 | f | 338 | 16.43 ± 10.53 | 13.98 ± 8.86 | 17.46 ± 9.82 | 16.64 ± 8.67 |
|  | m | 295 | 19.30 ± 12.33 | 16.49 ± 10.68 | 17.82 ± 9.93 | 16.92 ± 8.82 |
| 30-39 | f | 520 | 16.97 ± 11.10 | 15.06 ± 9.81 | 18.48 ± 10.11 | 17.27 ± 9.04 |
|  | m | 437 | 16.99 ± 10.75 | 15.34 ± 9.34 | 16.74 ± 8.90 | 15.70 ± 8.03 |
| 40-49 | f | 734 | 18.73 ± 12.52 | 17.61 ± 11.67 | 18.71 ± 10.17 | 16.53 ± 8.55 |
|  | m | 487 | 18.95 ± 11.32 | 18.23 ± 10.93 | 16.99 ± 8.44 | 15.08 ± 7.55 |
| 50-59 | f | 1031 | 20.81 ± 12.33 | 18.74 ± 11.67 | 19.11 ± 9.83 | 16.88 ± 8.78 |
|  | m | 832 | 19.14 ± 12.07 | 18.17 ± 10.67 | 17.07 ± 9.13 | 14.89 ± 7.94 |
| 60-69 | f | 709 | 22.96 ± 13.82 | 21.06 ± 12.45 | 19.28 ± 10.04 | 16.90 ± 8.86 |
|  | m | 576 | 19.35 ± 11.91 | 18.28 ± 10.61 | 16.85 ± 8.91 | 14.59 ± 8.16 |
| 70-79 | f | 639 | 21.51 ± 13.36 | 20.81 ± 11.92 | 19.40 ± 10.61 | 16.28 ± 9.42 |
|  | m | 549 | 19.78 ± 12.29 | 18.91 ± 11.33 | 17.01 ± 8.96 | 14.14 ± 7.56 |
| 80-89 | f | 358 | 19.37 ± 11.81 | 19.05 ± 11.82 | 17.32 ± 9.21 | 14.63 ± 7.87 |
|  | m | 305 | 17.26 ± 10.29 | 16.69 ± 9.62 | 13.97 ± 7.64 | 11.72 ± 5.69 |
